# Counterfactual Bell-State Analysis

**DOI:** 10.1038/s41598-018-32928-8

**Published:** 2018-10-02

**Authors:** Fakhar Zaman, Youngmin Jeong, Hyundong Shin

**Affiliations:** Department of Electronic Engineering, Kyung Hee University, Yongin-si, 17104 Korea

## Abstract

The Bell-state analysis to distinguish between the four maximally entangled Bell states requires the joint measurement on entangled particles. However, spatially separated parties cannot perform the joint measurement. In this paper, we present a *counterfactual* Bell-state analysis based on the chained quantum Zeno effect. This counterfactual analysis not only enables us to perform a complete Bell-state analysis, but also enables spatially separated parties to distinguish between the four Bell states without transmitting any physical particle over the channel.

## Introduction

Entanglement is one of the most fascinating resource for remarkable tasks in quantum information processing including quantum computing, quantum communication, and quantum cryptography^1–3^. The power of quantum protocols is enhanced by multiple degrees of freedom—known as *hyperentanglement*—realized in a multi-particle system^4–6^. For a bipartite system, the Bell-state analysis is a crucial step in various quantum communication protocols such as superdense coding^7,8^, which allows us to transmit two classical bits using a single qubit, and quantum teleportation^9,10^, which allows us to transmit an unknown qubit via a classical channel. To take full advantage of these protocols, one needs to be able to perform the complete Bell-state analysis. However, it has been proven that the complete Bell-state analysis is impossible using only linear operations^11–13^. The maximum achievable efficiency for the linear Bell-state analyzer is 50%^12^.

Hyperentanglement can be used for the complete Bell-state analysis^14–16^. Unlike the hyperentanglement assisted Bell-state analysis, a different approach^17^ has been proposed using the *quantum Zeno effect*^18^. The quantum Zeno effect is the inhibition between quantum states by frequent multiple measurements of the state, that is, the quantum state usually collapses back to the initial state if the time between measurements is short enough^19,20^. If spatially separated parties have an entangled pair and they perform the Bell-state analysis using the quantum Zeno effect, the probability of finding the particle over the quantum channel is one.

To date, all the proposed Bell-state analyzers for spatially separated parties need to send the physical particle over the channel. Here a question arises: “*Can spatially separated parties perform the Bell-state analysis without transmitting any physical particle over the channel*?” Surprisingly, the answer is *Yes*.

Counterfactual quantum communication^21^ is a surprising phenomenon that results from the quantum mechanics, which indicates that communication tasks can be achieved without transmitting any physical particle between two parties. The concept of *counterfactuality* originated from the interaction-free measurement^22^. The basic idea was to infer the presence of an absorptive object without interacting with it. The maximum achievable efficiency was limited by the margin of 50%. This efficiency was further improved to 100% by using the quantum Zeno effect^18^. Later, this idea has been extended to counterfactual quantum cryptography^23^. Subsequently, counterfactual quantum key distribution schemes and their security have been studied in detail^24–26^.

The protocol for direct counterfactual quantum communication based on the chained quantum Zeno effect (CQZE) has been proposed, which allows classical bits to be transferred between two parties without transmitting any particle between them^21^ (SLAZ13). Recently, the concept of counterfactual quantum communication is further expanded to the counterfactual entanglement distribution^27–29^ and counterfactual quantum information transfer^30,31^. In this work, we propose a counterfactual scheme for the complete Bell-state analysis based on the CQZE. We use the counterfactual CNOT gate^32^ (CCNOT gate) followed by the Hadamard gate to distinguish between the four Bell states. The proposed counterfactual scheme can perform the complete Bell-state analysis, but no physical particle passes over the channel.

The rest of the paper is organized as follows. In the next section, we first explain the operation of the CCNOT gate followed by counterfactual Bell-state analysis. In Sec. Discussion, we discuss our results under the non-asymptotic limits. At the end, we analyze the effect of the channel noise and discuss the experimental feasibility of our scheme.

## Results

### Counterfactual CNOT Gate

**Figure 1:**
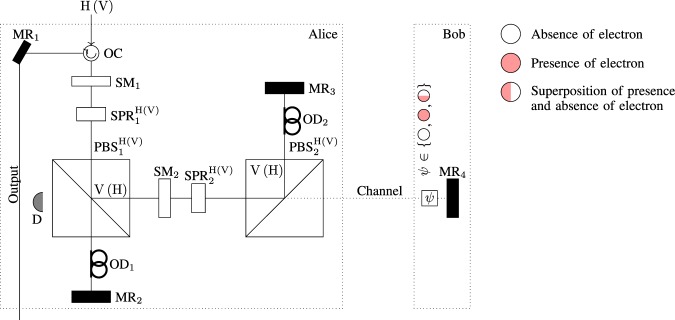
Michelson H(V)-CQZE setup. PBS stands for a polarizing beam splitter, MR stands for a mirror, OD for an optical delay, and OC for an optical circulator. The switchable mirror SM_1(2)_ is initially turned off to allow passing the photon, and once the photon is passed it will be turned on. After *M*(*N*) cycles, SM_1(2)_ is turned off again allowing the photon out. The switchable polarizing rotator SPR_1(2)_ rotates the polarization by small angle ${\theta }_{M(N)}=\frac{\pi }{2M(N)}$ once in each cycle $({{\rm{SM}}}_{1(2)}\to {{\rm{PBS}}}_{1(2)}^{{\rm{H}}({\rm{V}})})$. At Bob’s end, Bob either allows the photon to pass or block the channel by introducing an electron as a quantum absorptive object. Full counterfactulity is ensured as any photon found in the transmission channel would either absorbed by the electron or else end up at the detector D. Table 1 shows the operation of the H(V)-CQZE gate.

We consider the Michelson version of CQZE^24,28^ setup as shown in Fig. 1, where H(V) denotes the horizontal (vertical) polarization. Initially, the switchable mirror SM_1(2)_ is switched off to allow Alice’s photon in before being switched on again for *M*(*N*) outer (inner) cycles. The switchable polarization rotator ${{\rm{SPR}}}_{1(2)}^{{\rm{H}}({\rm{V}})}$ applies the following rotation on Alice’s photon before it is switched off for the rest of this cycle:

1 $${{\rm{SPR}}}_{1(2)}^{{\rm{H}}}\{\begin{array}{l}|{\rm{H}}\rangle \to \,\cos \,{\theta }_{i}\,|{\rm{H}}\rangle +\,\sin \,{\theta }_{i}\,|{\rm{V}}\rangle ,\\ |{\rm{V}}\rangle \to \,\cos \,{\theta }_{i}\,|{\rm{V}}\rangle -\,\sin \,{\theta }_{i}\,|{\rm{H}}\rangle ,\end{array}$$

2 $${{\rm{SPR}}}_{1(2)}^{{\rm{V}}}\{\begin{array}{l}|{\rm{V}}\rangle \to \,\cos \,{\theta }_{i}\,|{\rm{V}}\rangle +\,\sin \,{\theta }_{i}\,|{\rm{H}}\rangle ,\\ |{\rm{H}}\rangle \to \,\cos \,{\theta }_{i}\,|{\rm{H}}\rangle -\,\sin \,{\theta }_{i}\,|{\rm{V}}\rangle ,\end{array}$$

where ${\theta }_{i}=\pi /(2i)$ with $i=M$ for outer cycles and $i=N$ for inner cycles, respectively.

The Michelson CQZE setup takes H(V) photons as an input, with the polarizing beam splitter ${{\rm{PBS}}}_{\mathrm{1(2)}}^{{\rm{H}}({\rm{V}})}$ passing H(V) photons and reflecting V(H) as shown in Fig. 1. There are two possible scenarios,

1. If Bob allows the photon to pass, the inner cycles act as an obstacle for outer cycles. After *M* outer cycles, the photon will end up in the state $|{\rm{H}}({\rm{V}})\rangle$. If the photon is found in the transmission channel it will end up at the detector D. For large values of *M* and *N*, the probability of finding the photon in the transmission channel approaches to zero.
2. In the case where Bob blocks the channel, the inner cycles act as non-blocking for outer cycles. Unless the photon is absorbed by the absorptive object, the photon will be in the state $|{\rm{V}}({\rm{H}})\rangle$ after *M* outer cycles.

Table 1 shows the overall action of the I-CQZE gate under the asymptotic limits of *M* and *N*, where I, ${{\rm{I}}}^{\perp }\in \{{\rm{H}},{\rm{V}}\}$.

**Table 1:** Operation table for I-CQZE gate under the asymptotic limits of *M* and *N*, where I, ${{\rm{I}}}^{\perp }\in \{{\rm{H}},\,{\rm{V}}\}$.

Control State (electron)	Target State (photon)	Output State (photon)
${|{\rm{pass}}\rangle }_{{\rm{e}}}$	${|{\rm{I}}\rangle }_{{\rm{p}}}$	${|{\rm{I}}\rangle }_{{\rm{p}}}$
	${|{{\rm{I}}}^{\perp }\rangle }_{{\rm{p}}}$	—
${|{\rm{block}}\rangle }_{{\rm{e}}}$	${|{\rm{I}}\rangle }_{{\rm{p}}}$	${|{{\rm{I}}}^{\perp }\rangle }_{{\rm{p}}}$
	${|{{\rm{I}}}^{\perp }\rangle }_{{\rm{p}}}$	${|{\rm{I}}\rangle }_{{\rm{p}}}$

**Figure 2:**
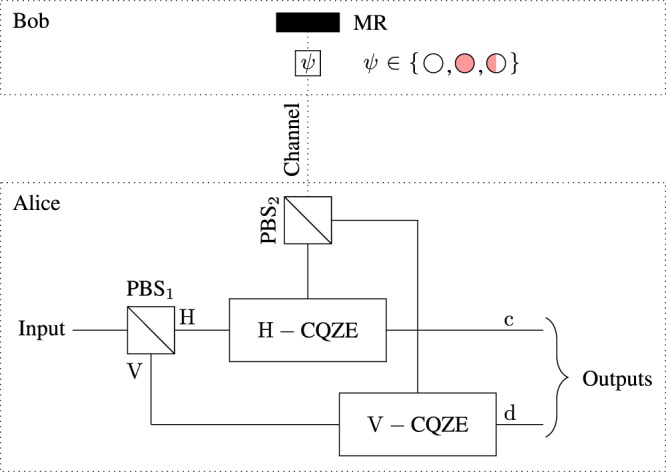
Counterfactual CNOT gate based on H(V)-CQZE setup. Here H is the horizontal polarization and V is the vertical polarization. Alice starts by sending her photon from the left which is first separated into H and V components using PBS_1_. These components feed into the corresponding CQZE setup. At the output of the counterfactual CNOT gate, the polarization of the existing photon determines the state of the control bit (quantum absorptive object).

The basic idea behind the CCNOT gate is direct counterfactual quantum communication^21^ where the control bit acts as a quantum absorptive object that can be in the superposition of two orthogonal states as shown in Fig. 2. Alice first need to pass her photon through PBS_1_ in order to separate the H and V components. Then, these components feed into the corresponding CQZE setup. At Bob’s end, Bob can block the transmission channel or allow the photon to pass or in their superposition. Unless the photon is discarded in counterfactual quantum communication, the photon will be in path c or d, or in the superposition of c and d after *M* outer and *N* inner cycles depending on the input state of the photon and the control bit. As *M*, $N\to {\rm{\infty }}$, the probability that the photon is found in the transmission channel approaches to zero.

### Counterfactual Bell-state Analysis

**Figure 3:**
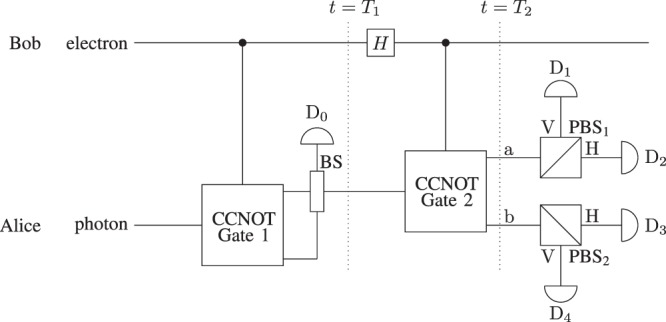
Counterfactual Bell-state analysis based on the chained quantum Zeno effect. Here BS stands for 50 : 50 beam splitter, the *H* stands for the Hadamard gate and the CCNOT gate is the counterfactual CNOT gate. By means of the CCNOT gate, Bell states can be analyzed without transmitting any physical particle over the channel where Bob’s entangled particle (electron) acts as a control bit. Initially the electron and photon are in entangled state. Alice starts the protocol by sending her photon towards the CCNOT gate. At *t* = *T*_1_, a polarization of the photon determines either the state: $|{{\rm{\Phi }}}^{\pm }\rangle$ or $|{{\rm{\Psi }}}^{\pm }\rangle$. After the Hadamard gate, Bob needs to transmit classical information $({|0\rangle }_{{\rm{e}}}\,{\rm{or}}\,{|1\rangle }_{{\rm{e}}})$ counterfactualy. For this, Alice send her photon in the CCNOT gate and the photon will be detected at one of the four detectors (D_1_, D_2_, D_3_, and D_4_) at *t* = *T*_2_. Each detector corresponds to one of the four Bell states which enables us to distinguish between the Bell states with certainty.

In the conventional Bell-state analysis, Alice uses the CNOT gate followed by the Hadamard gate (Bob’s side) on the entangled particles. Following the same procedure, we use the CCNOT gate^32^ to make our scheme counterfactual as shown in Fig. 3. The CCNOT gate is based on direct counterfactual quantum communication where quantum absorptive object act as control bit as shown in Fig. 2. In direct counterfactual quantum communication, the probability that the photon is found in the transmission channel for each of the Bob’s choices (allow the photon to pass or block the channel) depends on the number of *M* outer and *N* inner cycles of the CQZE. In the case photon is found in the transmission channel, the photon would either be absorbed by the absorptive object or ends up at detector D (see Fig. 1). This probability is asymptotically zero as *M* and *N* approach to infinity.

To demonstrate our scheme for counterfactual Bell-state analysis, we consider that Alice and Bob have an entangled pair of photon and electron, where the electron acts as the control bit and the photon acts as the target bit, respectively. Then, the Bell states are given by

3 $$|{{\rm{\Phi }}}^{\pm }\rangle =\frac{1}{\sqrt{2}}{(|{\rm{pass}}\rangle }_{{\rm{e}}}|{\rm{H}}{\rangle }_{{\rm{p}}}\pm |{\rm{block}}{\rangle }_{{\rm{e}}}|{\rm{V}}{\rangle }_{{\rm{p}}}),$$

4 $$|{{\rm{\Psi }}}^{\pm }\rangle =\frac{1}{\sqrt{2}}{(|{\rm{pass}}\rangle }_{{\rm{e}}}|{\rm{V}}{\rangle }_{{\rm{p}}}\pm |{\rm{block}}{\rangle }_{{\rm{e}}}|{\rm{H}}{\rangle }_{{\rm{p}}}),$$

where |pass〉_e_ = |0〉_e_, |block〉_e_ = |1〉_e_, |H〉_p_ = |0〉_p_, and |V〉_p_ = |1〉_p_; and the subscripts e and p denote the electron and photon, respectively.

Alice starts by sending her photon into the CCNOT gate. At *t* = *T*_1_, the photon will be horizontally polarized if the initial state is $|{{\rm{\Phi }}}^{\pm }\rangle$, while it will be vertically polarized if the initial state is $|{{\rm{\Psi }}}^{\pm }\rangle$. Then, the photon and electron will be in the separable state, and the Bell states in (3) and (4) transform into

5 $$|{{\rm{\Phi }}}^{\pm }\rangle \to \frac{1}{\sqrt{2}}{(|{\rm{pass}}\rangle }_{{\rm{e}}}\pm |{\rm{block}}{\rangle }_{{\rm{e}}})|{\rm{H}}{\rangle }_{{\rm{p}}},$$

6 $$|{{\rm{\Psi }}}^{\pm }\rangle \to \frac{1}{\sqrt{2}}{(|{\rm{pass}}\rangle }_{{\rm{e}}}\pm |{\rm{block}}{\rangle }_{{\rm{e}}})|{\rm{V}}{\rangle }_{{\rm{p}}}\mathrm{.}$$

**Figure 4:**
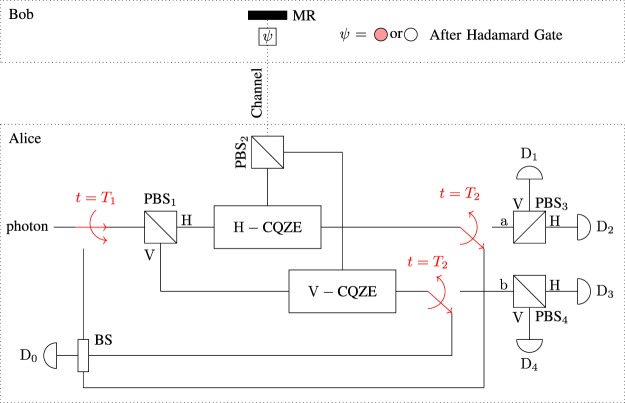
Feedback system of counterfactual Bell-state analysis using H(V)-CQZE setup. Alice starts by throwing her photon towards the PBS_1_ which separates H and V components of the input photon and feed them into corresponding CQZE setup. At *t* = *T*_1_, Alice throws her photon again towards PBS_1_ and uses the feedback system to determine the state of the electron after the Hadamard gate. Alice will measure the path of the photon at *t* = *T*_2_ to estimate the initial state.

After the Hadamard gate, Alice needs to determine the state of the electron, either present or absent, to distinguish between $|{{\rm{\Phi }}}^{+}\rangle (|{{\rm{\Psi }}}^{+}\rangle )$ and $|{{\rm{\Phi }}}^{-}\rangle (|{{\rm{\Psi }}}^{-}\rangle )$. To make our scheme fully counterfactual, we use a *feedback system* to determine the absence or presence of the electron as shown in Fig. 4. At *t* = *T*_2_, the photon is either in path a or b. If the photon is in path a, the initial state is $|{{\rm{\Phi }}}^{\pm }\rangle$, while if the photon is in path b, the initial state is $|{{\rm{\Psi }}}^{\pm }\rangle$. The polarization of the photon determines either the state x^+^ or the state x^−^ where ${\rm{x}}\in \{{\rm{\Phi }},{\rm{\Psi }}\}$. The photon will be detected by one of the four detectors. Each detector corresponds to one of the four Bell states which enables us to distinguish between the four Bell states with certainty. Table 2 shows the estimated initial state corresponding to each detector.

**Table 2:** Bell-state analysis under the asymptotic limits of *M* and *N*.

*t* = *T*_2_			
Photon Path	Photon Polarization State	Detector Clicks	Estimated Initial State
a	|V〉	D_1_	|Φ^−^〉
	|H〉	D_2_	|Φ^+^〉
b	|H〉	D_3_	|Ψ^−^〉
	|V〉	D_4_	|Ψ^+^〉

From the counterfactual Bell-state analysis in the paper, we conclude the following observations and remarks.

1. *Completeness:* The complete Bell-state analysis is itself a challenge. From Table 2, the counterfactual Bell-state analysis in this paper enables us to distinguish between the four Bell states without using the hyperentanglement. It is verified for finite numbers of *M* outer and *N* inner cycles for the CQZE (see Sec. Discussion).
2. *Counterfacuality:* We challenged the long-lasting assumption that spatially separated parties cannot perform the Bell-state analysis without transmitting any physical particle over the channel. In our scheme, the probability of finding the photon in the transmission channel approaches to zero under the asymptotic limits of *M* and *N*. Even for finite values of *M* and *N*, if the photon is found in the transmission channel either it will be absorbed by the electron or it will be discarded at detector D (see Fig. 1); and no detector will click. Unless the photon is discarded, one of the four detectors click which enables spatially separated parties to perform the complete Bell-state analysis without transmitting any physical particle over the channel.
3. *Resource Efficiency:* Quantum superdense coding is a prime application to utilize shared entanglement between two parties. In general, both of the entangled particles get destroyed at the end of the protocol to decode a two-bit classical message. Counterfactual Bell-state analysis enables spatially separated parties to implement quantum superdense coding without transmitting any physical particle over the channel at the cost of only one particle. As shown in Fig. 3, only the photon is absorbed by the detector to estimate the initial state.

## Discussion

In the previous section, we discussed the counterfactual Bell-state analysis under the ideal scenario. In this section, we show that counterfactual Bell-state analysis can be true: i) for finite values of *M* and *N*, and ii) in the presence of channel noise. Then, we further discuss the experimental feasibility for our counterfactual Bell-state analysis.

### Finite Values of *M* and *N*

We first show that all results are also true for finite values of *M* and *N*. For the finite values of *M* and *N*, the counterfactuality of the SLAZ13 has been analyzed^33,34^. It was shown that the protocol can be counterfactual only for one value of the transmitted bit $({|1\rangle }_{{\rm{e}}})$.

Recently, experimental realization^35^ of SLAZ13^21^ has been presented to preserve the counterfactual property for both values of the transmitted bit via the CQZE setup using the single-photon source. In the case of multi-photon source or coherent state light, the counterfactuality is not ensured if Bob allows the photon to pass $({|0\rangle }_{{\rm{e}}})$. In the presence of the absorptive object, the amplitude of the coherent state which is found in the transmission channel will be absorbed by the absorptive object and no detector clicks. In the absence of the absorptive object, the amplitude of the coherent state found in the transmission channel is nonzero even when the detector D does not click (see Fig. 1) which violates the counterfactuality of the protocol. To ensure the couterfactuality for both values of the transmitted bit for finite values of *M* and *N*, they used the single-photon source. The overall action of the modified SLAZ13 scheme is same as shown in Table 1. The only difference is that there exists a success probability corresponding to each Bob’s choice, either block the transmission channel or allow the photon to pass. These probabilities are given by^21^

7 $${P}_{{\rm{pass}}}={\cos }^{(2M)}\,{\theta }_{M},$$

8 $${P}_{{\rm{block}}}=|{y}_{\{M,0\}}{|}^{2},$$

where ${\theta }_{j}=\pi /(2j)$ for $j\in \{M,N\}$; *P*_pass_ is the success probability when Bob allows the photon to pass while *P*_block_ is the success probability where Bob blocks the photon path; and ${y}_{\{M,0\}}$ can be obtained from the recursion relations:

9 $${y}_{\{m+1,0\}}={b}_{M}{x}_{m}+{a}_{M}{y}_{\{m,N\}},$$

10 $${x}_{m+1}={a}_{M}{x}_{m}-{b}_{M}{y}_{\{m,N\}},$$

11 $${y}_{\{m,n\}}={a}_{N}{y}_{\{m,n-1\}},$$

where ${x}_{1}={a}_{M}$, ${y}_{\{1,0\}}={b}_{M}$, ${a}_{j}=\,\cos \,{\theta }_{j}$, and ${b}_{j}=\,\sin \,{\theta }_{j}$ for $j\in \{M,N\}$.

Similarly, the required condition to preserve the counterfactual property of our scheme for the finite values of *M* and *N* is that the single-photon source must be used at the time of entanglement distribution between Alice and Bob. To explain the transformation of (3) and (4), let $|\cdot \rangle \mathop{\longrightarrow }\limits_{m,n}|\cdot \rangle$ denote the state transformation after *m* (<*M*) outer and *n* (<*N*) inner cycles. Then, we have

12 $$\begin{array}{lll}|{\rm{pass}}{\rangle }_{{\rm{e}}}|{\rm{I}}{\rangle }_{{\rm{p}}} & \mathop{\mathop{\longrightarrow }\limits^{{{\rm{SPR}}}_{1}}}\limits_{1,0} & |{\rm{pass}}{\rangle }_{{\rm{e}}}{(\cos {\theta }_{M}|{\rm{I}}\rangle }_{{\rm{p}}}+\,\sin \,{\theta }_{M}|{{\rm{I}}}^{\perp }{\rangle }_{{\rm{p}}})\\  & \mathop{\mathop{\longrightarrow }\limits^{{{\rm{SPR}}}_{2}}}\limits_{1,1} & |{\rm{pass}}{\rangle }_{{\rm{e}}}{(\cos {\theta }_{M}|{\rm{I}}\rangle }_{{\rm{p}}}+\,\sin \,{\theta }_{M}{(\cos {\theta }_{N}|{{\rm{I}}}^{\perp }\rangle }_{{\rm{p}}}-\,\sin \,{\theta }_{N}|{\rm{I}}{\rangle }_{{\rm{p}}}))\\  & \mathop{\mathop{\longrightarrow }\limits^{{{\rm{PBS}}}_{1}}}\limits_{1,N} & |{\rm{pass}}{\rangle }_{{\rm{e}}}{(\cos {\theta }_{M}|{\rm{I}}\rangle }_{{\rm{p}}})\\  & \mathop{\mathop{\longrightarrow }\limits^{{{\rm{SPR}}}_{1}}}\limits_{2,0} & |{\rm{pass}}{\rangle }_{{\rm{e}}}\,\cos \,{\theta }_{M}{(\cos {\theta }_{M}|{\rm{I}}\rangle }_{{\rm{p}}}+\,\sin \,{\theta }_{M}|{{\rm{I}}}^{\perp }{\rangle }_{{\rm{p}}})\\  & \mathop{\mathop{\longrightarrow }\limits^{{{\rm{SPR}}}_{1}}}\limits_{m,0} & |{\rm{pass}}{\rangle }_{{\rm{e}}}\,{\cos }^{m-1}\,{\theta }_{M}{(\cos {\theta }_{M}|{\rm{I}}\rangle }_{{\rm{p}}}+\,\sin \,{\theta }_{M}|{{\rm{I}}}^{\perp }{\rangle }_{{\rm{p}}}),\end{array}$$

13 $$\begin{array}{lll}|{\rm{block}}{\rangle }_{{\rm{e}}}|{\rm{I}}{\rangle }_{{\rm{p}}} & \mathop{\mathop{\longrightarrow }\limits^{{{\rm{PBS}}}_{1}}}\limits_{1,N} & |{\rm{block}}{\rangle }_{{\rm{e}}}{(\cos {\theta }_{M}|{\rm{I}}\rangle }_{{\rm{p}}}+\,\sin \,{\theta }_{M}|{{\rm{I}}}^{\perp }{\rangle }_{{\rm{p}}})\\  & \mathop{\mathop{\longrightarrow }\limits^{{{\rm{SPR}}}_{1}}}\limits_{2,0} & |{\rm{block}}{\rangle }_{{\rm{e}}}{(\cos 2{\theta }_{M}|{\rm{I}}\rangle }_{{\rm{p}}}+\,\sin \,2{\theta }_{M}|{{\rm{I}}}^{\perp }{\rangle }_{{\rm{p}}})\\  & \mathop{\mathop{\longrightarrow }\limits^{{{\rm{SPR}}}_{1}}}\limits_{m,0} & |{\rm{block}}{\rangle }_{{\rm{e}}}{(\cos m{\theta }_{M}|{\rm{I}}\rangle }_{{\rm{p}}}+\,\sin \,m{\theta }_{M}|{{\rm{I}}}^{\perp }{\rangle }_{{\rm{p}}}),\end{array}$$

for I, ${{\rm{I}}}^{\perp }\in \{{\rm{H}},\,{\rm{V}}\}$ and *N* is assumed to be large such that ${\cos }^{N}\,{\theta }_{N}\approx 1$. After *M* outer and *N* inner cycles, the photon and electron will be in the separable state, which is given by

14 $$|{{\rm{\Phi }}}^{\pm }\rangle \mathop{\mathop{\longrightarrow }\limits^{{{\rm{SM}}}_{1}}}\limits_{M,N}\frac{1}{\sqrt{2}}(\sqrt{{P}_{{\rm{pass}}}}|{\rm{pass}}{\rangle }_{{\rm{e}}}\pm \sqrt{{P}_{{\rm{block}}}}|{\rm{block}}{\rangle }_{{\rm{e}}})|{\rm{H}}{\rangle }_{{\rm{p}}},$$

15 $$|{{\rm{\Psi }}}^{\pm }\rangle \mathop{\mathop{\longrightarrow }\limits^{{{\rm{SM}}}_{1}}}\limits_{M,N}\frac{1}{\sqrt{2}}(\sqrt{{P}_{{\rm{pass}}}}|{\rm{pass}}{\rangle }_{{\rm{e}}}\pm \sqrt{{P}_{{\rm{block}}}}|{\rm{block}}{\rangle }_{{\rm{e}}})|{\rm{V}}{\rangle }_{{\rm{p}}}\mathrm{.}$$

Note that (14) and (15) are not orthonormal because the probability that the photon is discarded in the counterfactual quantum communication is nonzero for the finite values of *M* and *N*.

From (12) and (13), the probability that the photon is not discarded, denoted by *P*_s_, till *t* = *T*_1_ for any input Bell state is given by^32^

16 $${P}_{{\rm{s}}}={(1-\frac{1}{2}{\sin }^{2}{\theta }_{M})}^{M}\,\prod _{m=1}^{M}\,{(1-\frac{1}{2}{\sin }^{2}m{\theta }_{M}{\sin }^{2}{\theta }_{N})}^{N}\mathrm{.}$$

Unless the photon is discarded, the photon will be detected at one of the four detectors at *t* = *T*_2_, and we can estimate the initial state with the probability one. The success probabilities ${P}_{{{\rm{D}}}_{1}}$, ${P}_{{{\rm{D}}}_{2}}$, ${P}_{{{\rm{D}}}_{3}}$, and ${P}_{{{\rm{D}}}_{4}}$ for the corresponding initial Bell states $|{{\rm{\Phi }}}^{-}\rangle$, $|{{\rm{\Phi }}}^{+}\rangle$, $|{{\rm{\Psi }}}^{-}\rangle$, and $|{{\rm{\Psi }}}^{+}\rangle$ are respectively given by

17 $${P}_{{{\rm{D}}}_{1}}={P}_{{{\rm{D}}}_{3}}={P}_{{\rm{s}}}\,\prod _{m=1}^{M}\,{(1-{\sin }^{2}m{\theta }_{M}{\sin }^{2}{\theta }_{N})}^{N},$$

18 $${P}_{{{\rm{D}}}_{2}}={P}_{{{\rm{D}}}_{4}}={P}_{{\rm{s}}}{(1-{\sin }^{2}{\theta }_{M})}^{M}\mathrm{.}$$

**Figure 5:**
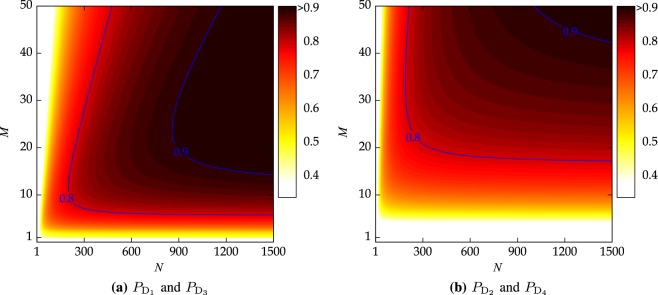
Success probabilities ${P}_{{{\rm{D}}}_{1}}$, ${P}_{{{\rm{D}}}_{2}}$, ${P}_{{{\rm{D}}}_{3}}$, and ${P}_{{{\rm{D}}}_{4}}$ as a function of (*M*, *N*) under the ideal channel conditions.

We plot the success probabilities against the different values of *M* and *N* in Fig. 5 under the ideal channel conditions. The state transition matrix for each Bell state and the detectors is given by

19 $$T=\begin{array}{cc}{{\rm{D}}}_{1}\,\,{{\rm{D}}}_{2}\,\,{{\rm{D}}}_{3}\,\,{{\rm{D}}}_{4} & \\ (\begin{array}{cccc}{P}_{{{\rm{D}}}_{1}} & 0 & 0 & 0\\ 0 & {P}_{{{\rm{D}}}_{2}} & 0 & 0\\ 0 & 0 & {P}_{{{\rm{D}}}_{3}} & 0\\ 0 & 0 & 0 & {P}_{{{\rm{D}}}_{4}}\end{array}) & \begin{array}{l}|{{\rm{\Phi }}}^{-}\rangle \\ |{{\rm{\Phi }}}^{+}\rangle \\ |{{\rm{\Psi }}}^{-}\rangle \\ |{{\rm{\Psi }}}^{+}\rangle .\end{array}\end{array}$$

From (19), we conclude that even for finite values of *M* and *N*, we can distinguish between the four Bell states with the *probability one* unless the photon is discarded in counterfactual quantum communication.

### Effect of Channel Noise

We now analyze the effect of channel noise under the asymptotic limits of *M* and *N*. Let *p* be the *blocking probability* caused by the noise in the quantum channel between Alice and Bob at the CCNOT gate. In the protocol, the blocking event does not change the output at Alice’s side if Bob blocks the photon path. However, it causes the problem if Bob allows the photon to pass but the blocking event is occurred due to the channel noise. To explain this effect of the channel noise, we first consider the states $|{{\rm{\Phi }}}^{\pm }\rangle$ given in (3). The state transformations of the electron and the photon can be modified as

20 $$\begin{array}{lll}|{{\rm{\Phi }}}^{\pm }\rangle  & \underset{{\rm{Gate}}\,{\rm{1}}}{\overset{{\rm{CCNOT}}}{\longrightarrow }} & \frac{1}{\sqrt{2}}\{|{\rm{pass}}{\rangle }_{{\rm{e}}}(\sqrt{1-p}|{\rm{H}}{\rangle }_{{\rm{p}}}+\sqrt{p}|{\rm{V}}{\rangle }_{{\rm{p}}})+|{\rm{block}}{\rangle }_{{\rm{e}}}|{\rm{H}}{\rangle }_{{\rm{p}}}\}\end{array}$$

21 $$\begin{array}{lll} & \mathop{\longrightarrow }\limits^{{\rm{BS}}} & \sqrt{\frac{q}{2}}\{|{\rm{pass}}{\rangle }_{{\rm{e}}}(\sqrt{1-p}|{\rm{H}}{\rangle }_{{\rm{p}}}+\sqrt{p}|{\rm{V}}{\rangle }_{{\rm{p}}})+|{\rm{block}}{\rangle }_{{\rm{e}}}|{\rm{H}}{\rangle }_{{\rm{p}}}\}\end{array}$$

22 $$\begin{array}{lll} & \mathop{\longrightarrow }\limits^{H} & \frac{\sqrt{q}}{2}\{((\sqrt{1-p}\pm 1)|{\rm{pass}}{\rangle }_{{\rm{e}}}+(\sqrt{1-p}\mp 1)|{\rm{block}}{\rangle }_{{\rm{e}}})|{\rm{H}}{\rangle }_{{\rm{p}}}\\  &  & +\sqrt{p}{(|{\rm{pass}}\rangle }_{{\rm{e}}}+|{\rm{block}}{\rangle }_{{\rm{e}}})|{\rm{V}}{\rangle }_{{\rm{p}}}\},\end{array}$$

where $q=\frac{{(1+\sqrt{1-p})}^{2}+p}{4}$ is the probability that the photon is not discarded at the detector D_0_. Similarly, after the Hadamard gate, the initial Bell states $|{{\rm{\Psi }}}^{\pm }\rangle$ given in (4) are transformed into

23 $$\begin{array}{rcl}|{{\rm{\Psi }}}^{\pm }\rangle  & \to  & \frac{\sqrt{q}}{2}\{((\sqrt{1-p}\pm 1)|{\rm{pass}}{\rangle }_{{\rm{e}}}+(\sqrt{1-p}\mp 1)|{\rm{block}}{\rangle }_{{\rm{e}}})|{\rm{V}}{\rangle }_{{\rm{p}}}\\  &  & +\,\sqrt{p}{(|{\rm{pass}}\rangle }_{{\rm{e}}}+|{\rm{block}}{\rangle }_{{\rm{e}}})|{\rm{H}}{\rangle }_{{\rm{p}}}\}.\end{array}$$

**Figure 6:**
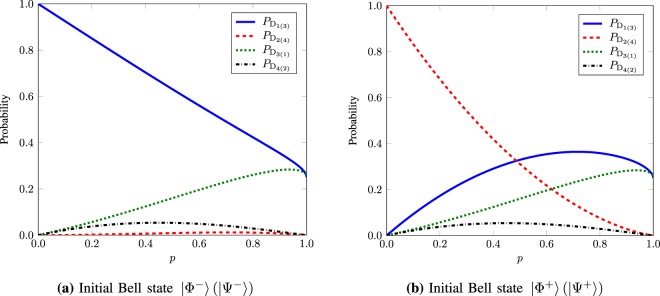
Probabilities ${P}_{{{\rm{D}}}_{1(3)}}$, ${P}_{{{\rm{D}}}_{2(4)}}$, ${P}_{{{\rm{D}}}_{3(1)}}$, and ${P}_{{{\rm{D}}}_{4(2)}}$ as a function of *p* for the corresponding input Bell states (**a**) |Φ^−^〉 (|Ψ^−^〉) and (**b**) |Φ^+^〉 (|Ψ^+^〉) under the asymptotic limits of *M* and *N*.

At *t* = *T*_2_, the state transformation matrix $\tilde{T}$ for each Bell state and the detectors is given by

24 $$\tilde{T}=\frac{1}{16}({{\rm{A}}}_{+}+p)\begin{array}{cc}{{\rm{D}}}_{1}\,\,\,\,\,{{\rm{D}}}_{2}\,\,\,\,\,{{\rm{D}}}_{3}\,\,\,\,\,{{\rm{D}}}_{4} & \\ (\begin{array}{cccc}p{{\rm{A}}}_{-}+{{\rm{A}}}_{+} & {{\rm{A}}}_{-}(1-p) & p+{p}^{2} & p-{p}^{2}\\ p{{\rm{A}}}_{+}+{{\rm{A}}}_{-} & {{\rm{A}}}_{+}(1-p) & p+{p}^{2} & p-{p}^{2}\\ p+{p}^{2} & p-{p}^{2} & p{{\rm{A}}}_{-}+{{\rm{A}}}_{+} & {{\rm{A}}}_{-}(1-p)\\ p+{p}^{2} & p-{p}^{2} & p{{\rm{A}}}_{+}+{{\rm{A}}}_{-} & {{\rm{A}}}_{+}(1-p)\end{array}) & \begin{array}{l}|{{\rm{\Phi }}}^{-}\rangle \\ |{{\rm{\Phi }}}^{+}\rangle \\ |{{\rm{\Psi }}}^{-}\rangle \\ |{{\rm{\Psi }}}^{+}\rangle ,\end{array}\end{array}$$

where ${{\rm{A}}}_{\pm }={(\sqrt{1-p}\pm 1)}^{2}$. In Fig. 6, we plot the probabilities ${P}_{{{\rm{D}}}_{1(3)}}$, ${P}_{{{\rm{D}}}_{2(4)}}$, ${P}_{{{\rm{D}}}_{3(1)}}$, and ${P}_{{{\rm{D}}}_{4(2)}}$ as a function of *p* for the corresponding input Bell states (a) $|{{\rm{\Phi }}}^{-}\rangle (|{{\rm{\Psi }}}^{-}\rangle )$ and (b) $|{{\rm{\Phi }}}^{+}\rangle (|{{\rm{\Psi }}}^{+}\rangle )$ under the asymptotic limits of *M* and *N*.

### Experimental Feasibility

For an experimental realization of counterfactual Bell-state analysis, there are two major problems to be concerned. The first one is to guarantee the phase stability of the CQZE gate which is the building block of our scheme as shown in Fig. 4. The practical realization of quantum counterfactual-like communication has been recently demonstrated based on the single-photon source^35^ and the weak coherent light^36^.

**Figure 7:**
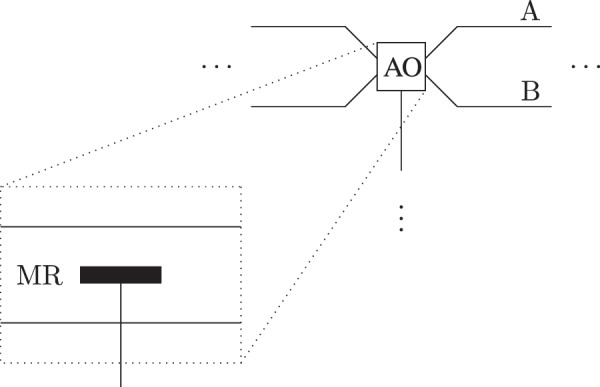
Bob’s device for experiment design. AO stands for an absorptive object (electron). A and B are upper and lower paths, respectively. If the electron in the path A, it does not interact with the photon, which shows the absence of the absorptive object and the photon will be reflected by the mirror MR. In case the electron is in the path B, it will absorb the photon if it is found in the transmission channel.

Another problem in the experimental realization is to generate a superposition state of the absorptive object. In our scheme, we used the electron as the quantum absorptive object which can take the superposition of two paths. To overcome the problem of superposition of presence and absence, we introduce a mirror between two paths as shown in Fig. 7. If the electron is in the path A, it shows the absence of the absorptive object. In case the electron is in the path B, the absorptive object is blocking the transmission channel.
